# Both ATM and DNA-PK Are the Main Regulators of HIV-1 Post-Integrational DNA Repair

**DOI:** 10.3390/ijms24032797

**Published:** 2023-02-01

**Authors:** Andrey Anisenko, Anastasiia Nefedova, Yulia Agapkina, Marina Gottikh

**Affiliations:** 1 Chemistry Department, Lomonosov Moscow State University, 119992 Moscow, Russia; 2 Faculty of Bioengineering and Bioinformatics, Lomonosov Moscow State University, 119992 Moscow, Russia; 3 Belozersky Institute of Physico-Chemical Biology, Lomonosov Moscow State University, 119992 Moscow, Russia

**Keywords:** HIV-1, DNA-PK, ATM, Ku70, integrase, post-integrational repair

## Abstract

The integration of a DNA copy of an HIV-1 RNA genome into the host genome, carried out by the viral enzyme integrase, results in the formation of single-stranded gaps in cellular DNA that must be repaired. Here, we have analyzed the involvement of the PI3K kinases, ATM, ATR, and DNA-PKcs, which are important players in the DNA damage response (DDR) in HIV-1 post-integrational DNA repair (PIR). The participation of the DNA-PK complex in HIV-1 PIR has been previously shown, and the formation of a complex between the viral integrase and the DNA-PK subunit, Ku70, has been found to be crucial for efficient PIR. Now, we have shown that the inhibition of both DNA-PKcs and ATM, but not ATR, significantly reduces PIR efficiency. The activation of both kinases is a sequential process, where one kinase, being activated, activates the other, and it occurs simultaneously with the integration of viral DNA. This fact suggests that the activation of both kinases triggers PIR. Most interestingly, the activation of not only DNA-PKcs, but also ATM depends on the complex formation between integrase and Ku70. The elucidation of the interactions between viruses and DDR is important both for understanding the modulation of host cell functions by these pathogens and for developing new approaches to combat viral infections.

## 1. Introduction

Viruses from the genus *Lentivirus* are unique among the Retroviridae family in their ability to infect both dividing and non-dividing cells. Their ability to integrate the DNA copy (cDNA) of the RNA genome is of great interest to researchers developing approaches to modify the cellular genome. The extensive research on the molecular aspects of *Lentivirus*, and especially HIV-1 replication, has led to the development of biosafe vectors that are now widely used not only in molecular biology, but also in clinical practice [1]. Third-generation, self-inactivating HIV-1 based vectors have recently been used in multiple clinical trials to introduce genes into hematopoietic stem cells to correct immunodeficiencies and hemoglobinopathies [2,3]. The introduction of a chimeric antigen receptor (CAR) into mature T cells using such vectors allows for the production of CAR-T cells, which are used to develop next generation therapies for cancer, HIV infection, and many other applications [4,5,6,7,8,9,10].

The development of all such approaches is impossible without deep knowledge of the molecular biology of HIV-1. It is well known that after the entry of the virus into the cell, viral reverse transcriptase synthesizes the cDNA of HIV-1 genomic RNA [11]. The preintegration complex (PIC), consisting of cDNA, viral proteins, including reverse transcriptase and integrase (IN), and many cellular proteins, approaches actively transcribed regions of the cellular genome through the interaction of the PIC component, LEDGF/p75, with epigenetic marks, such as H3K36me3. Then, IN inserts the viral cDNA into the cellular DNA [12]. This stage in most works is called the final step of early replication. However, there is another important, albeit less mentioned, step, post-integrational DNA repair (PIR).

The integration results in five nucleotide gaps that flank the integrated DNA and must be repaired prior to the transition from the early replicative stages to active viral transcription. The repair process is also necessary to remove unpaired overhanging dinucleotides at the 5′-ends of the integrated viral DNA. It has been generally implied that this step is performed by the cellular DNA repair machinery [13,14,15]. A screen of knockout libraries showed that proteins from numerous DNA repair pathways participate in HIV-1 replication, i.e., base and nucleotide excision, homologous recombination (HR), and non-homologous end joining (NHEJ) [16,17,18,19]; however, the lack of methodology for a PIR study did not allow the proteins from these repair systems and their role in PIR to be linked unequivocally.

In 2018, we described the first quantitative assay to measure PIR efficiency [20]. Using it, we demonstrated the participation of DNA-PKcs, which is the catalytic subunit of DNA-dependent protein kinase (DNA-PK), in this process [21]. It was rather surprising because DNA-PKcs is a major regulator of the DNA double-strand break (DSB) repair by the NHEJ pathway [22], but the HIV-1 integration intermediate does not contain DSBs. According to the model described in [21], the PIR process is initiated by the interaction of HIV-1 integrase (IN) with the cellular Ku70 protein, which is usually in a heterodimeric complex with the Ku80 protein. This interaction leads to the recruiting of DNA-PKcs and its subsequent activation. The activated DNA-PKcs recruits downstream proteins and regulates their activity through phosphorylation. These proteins need to be further identified.

DNA-PKcs, along with two other DNA-repair kinases, ATM and ATR, belongs to the PI3K-related kinase superfamily (PIKK) [22]. A possible role of DNA-PKcs in HIV-1 replication was first suggested in the works of R. Daniel et al., since the replication of VSV-G-pseudotyped HIV-based vector was reduced to 20% in DNA-PKcs-deficient pre-B lymphocyte from scid mice [23]. The incomplete decrease suggested that there might be an alternative kinase, possibly belonging to the PIKK family, which supported virus replication. This suggestion was supported by the fact that treatment of DNA-PKcs^−/−^ cells with wortmannin, an irreversible PIKK inhibitor, further reduced cell survival after virus transduction [24].

Further investigations showed that ATM-deficient cells were less efficiently infected with HIV-1-based pseudoviruses [24,25,26]. It was also shown that Ku-55933, a specific ATM inhibitor, efficiently suppressed HIV-1 infection. It decreased the levels of integrated DNA but did not affect the total amount of viral DNA [26]. However, it should be noted that the level of integrated DNA decreased with time: 24 h after infection, it was the same in Ku-55933-treated and control cells, whereas, after 48 h, the amount of integrated DNA dropped sharply in the treated cells and remained about the same in the control cell line [26]. Apparently, the cells where post-integration DNA damage could not be repaired die 24–48 h after infection, which explains the described observations. Although this indicates a possible involvement of ATM in HIV-1 PIR, direct evidence of this fact has not been available until now.

In this work, we analyzed the involvement of three kinases from the PIKK family in the regulation of PIR. Using specific inhibitors of DNA-PKcs, ATM, and ATR, as well as the qPCR assay to measure PIR efficiency [20], we determined that along with DNA-PKcs, ATM, but not ATR, is essential for PIR. We demonstrated that both kinases are activated in response to the integration of viral cDNA. ATM and DNA-PKcs act sequentially in the PIR process, and the activation of both kinases is required for HIV-1 replication. Most interestingly, the activation of both DNA-PKcs and ATM is dependent on the formation of the Ku70-IN complex, which may explain how the proteins are involved in PIR despite the absence of DSB in the integration intermediate.

## 2. Results

### 2.1. ATM and DNA-PKcs, but Not ATR, Are Essential for HIV-1 Post-Integrational Repair

**Figure 1:**
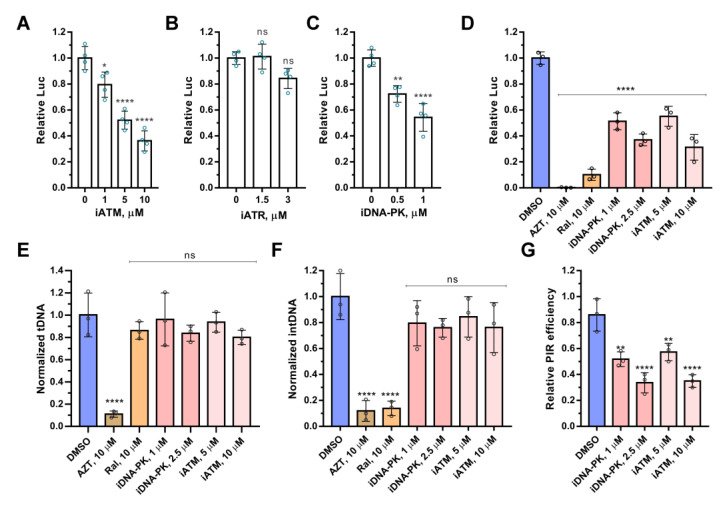
The effect of inhibitors of ATM, ATR, and DNA-PKcs on HIV-1 post-integrational repair. (**A**–**C**) Analysis of the relative luciferase expression in 293T cells transduced by HIV_wt in presence of increasing concentrations of Ku-55933 (iATM), AZ20 (iATR), and Nu7441 (iDNA-PK). (**D**) The effect of azidothymidine (AZT), raltegravir (Ral), iATM, and iDNA-PK on luciferase expression in 293T cells transduced by HIV_wt. (**E**,**F**) The level of the total viral DNA (**E**) and integrated viral DNA (**F**) in 293T cells transduced by HIV_wt in presence of AZT, Ral, iATM, and iDNA-PK. (**G**) Efficiency of post-integrational DNA repair in 293T cells transduced by HIV_wt in presence of iATM or iDNA-PK. Mean values ± SD of three (**D**–**G**) or four (**A**–**C**) independent experiments are presented. Significance was determined by one-way ANOVA with Dunnett’s correction for multiple comparisons, ns—not significant, * = adj. *p*-value < 0.05, ** = adj. *p*-value < 0.01, **** = adj. *p*-value < 0.0001.

To analyze the involvement of PIKK kinases in HIV-1 replication, we used their specific inhibitors: Nu7441 for DNA-PKcs (iDNA-PKcs), Ku-55933 for ATM (iATM), and AZ20 for ATR kinase (iATR). The inhibitors’ effect was tested on VSV-G-pseudotyped single-round luciferase-expressing HIV-based pseudovirus (here and below HIV_wt). HIV-1 transduction efficiency was quantified by measuring luciferase activity and normalized to cell viability (Figure S1). The inhibitors of DNA-PKcs and ATM, but not ATR, reduced luciferase expression in a dose-dependent manner after the transduction of HEK 293T by HIV_wt (Figure 1A–C).

Previously, iDNA-PKcs was demonstrated to inhibit post-integrational DNA repair without affecting the reverse transcription or integration process [21]. Here, we used the same strategy to decipher the role of the ATM kinase activity in HIV-1 replication.

We measured the level of total, integrated, and repaired viral DNA using the qPCR assays described in [20] in HEK 293T cells transduced by HIV_wt in the presence of iATM. Azidothymidine (AZT), raltegravir (Ral), and iDNA-PKcs were used as reference compounds, which inhibit reverse transcription, integration, or PIR, respectively (Figure 1D–G). All compounds negatively affected the luciferase expression from HIV_wt (Figure 1D), but due to the influence on different stages of virus replication. AZT reduced the amount of total HIV-1 DNA, i.e., the product of reverse transcription (Figure 1E), Ral inhibited integration of cDNA (Figure 1F), and both iATM and iDNA-PKcs blocked PIR without an influence on reverse transcription or integration processes (Figure 1E–G). Therefore, the kinase activity of ATM, as with DNA-PKcs, is required for HIV-1 PIR. If for DNA-PKcs, this was shown earlier [21], then for ATM, this is the first direct evidence of its participation in PIR.

### 2.2. ATM Activation Depends on the Formation of a Complex between HIV-1 Integrase and the Cellular Ku70 Protein

As is mentioned above, DNA-PKcs is involved in PIR due to the formation of the HIV-1 IN complex with Ku70, and therefore PIR can be suppressed by disrupting this complex [21]. It can be completed by entering the double substitution E212A/L213A into IN. An HIV-based pseudovirus containing this IN mutant (HIV_mut) transduced different cell lines, including PBMCs, 5–7 times worse than HIV_wt. At the same time, HIV_mut was insensitive to the DNA-PKcs inhibitor Nu7441 [21].

**Figure 2:**
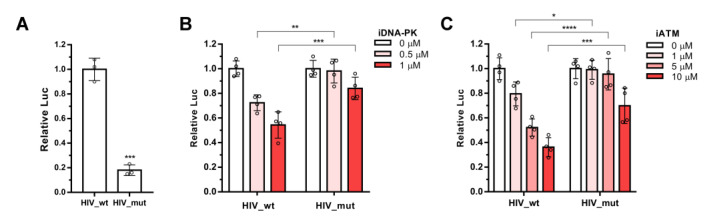
The effect of ATM and DNA-PKcs inhibitors on luciferase activity in 293T cells transduced by HIV_wt and HIV_mut. (**A**) HIV_mut transduced 293T cells five times worse than HIV_wt. Significance was determined by two-tailed Student’s *t*-test, *** = *p* < 0.001. (**B**,**C**) Analysis of the relative luciferase expression in 293T cells transduced by HIV_wt or HIV_mut in presence of increasing concentrations of Nu7441 (iDNA-PK) or Ku-55933 (iATM). Data for HIV_wt or HIV_mut were normalized to 0 μM points for each vector independently. Mean values ± SD of three (**A**) or four (**B**,**C**) independent experiments are presented. Significance was determined by two-way ANOVA with Sidak’s correction for multiple comparisons, * = adj. *p*-value < 0.05, ** = adj. *p*-value < 0.01, *** = adj. *p*-value < 0.001, **** = adj. *p*-value < 0.0001.

We decided to analyze whether ATM activation depends on the IN binding with Ku70, as was shown for DNA-PKcs. 293T cells were transduced by HIV_wt or HIV_mut in the presence of increasing concentrations of iATM or iDNA-PKcs as a positive control. Luciferase activity was measured in the transduced cells 24 h post transduction. Data obtained for these cells were normalized to luciferase levels in cells transduced with HIV_wt or HIV_mut without inhibitor added to make the results easier to interpret. It should be noted that in our experiments, HIV_mut transduced 293T cells at a level of 18% of HIV_wt (Figure 2A). Consistent with results previously published [21], iDNA-PK inhibited cell transduction by HIV_wt, and had no significant influence on HIV_mut. Only at the highest concentration tested, iDNA-PK reduced HIV_mut luciferase expression by 16%, while HIV_wt was inhibited by 55% (Figure 2B). In the case of ATM inhibitor Ku-55933, similar effects were observed (Figure 2C). iATM inhibited HIV_wt in a dose-dependent manner, and had no effect on the HIV_mut luciferase expression at 1 or 5 μM concentrations; at 10 μM, it inhibited HIV_mut by 35%, but, statistically, it was less than for HIV_wt (69%) (Figure 2C). Similar results were obtained with the ATM inhibitor Ku-60019, which has a higher solubility (Figure S2). Therefore, the activation of both ATM and DNA-PKcs during HIV-1 PIR is dependent on the formation of the IN-Ku70 complex.

### 2.3. Integration of Viral DNA Is a Signal for ATM and DNA-PKcs Activation

We have previously demonstrated that there is a delay of up to 10 h between the formation of integrated and repaired forms of viral DNA [20]. DNA-PKcs and ATM activation can occur at any time during this period. To accurately determine the activation kinetics for both kinases, we performed a time-of-addition experiment. This approach determines how long the addition of a compound can be postponed before it loses its antiviral activity [27]. Indeed, if an inhibitor that interferes with, say, viral reverse transcriptase is present while the reverse transcription process is occurring, it can inhibit viral replication. Conversely, if this inhibitor is added after reverse transcription is complete, it will no longer be effective in blocking viral replication.

**Figure 3:**
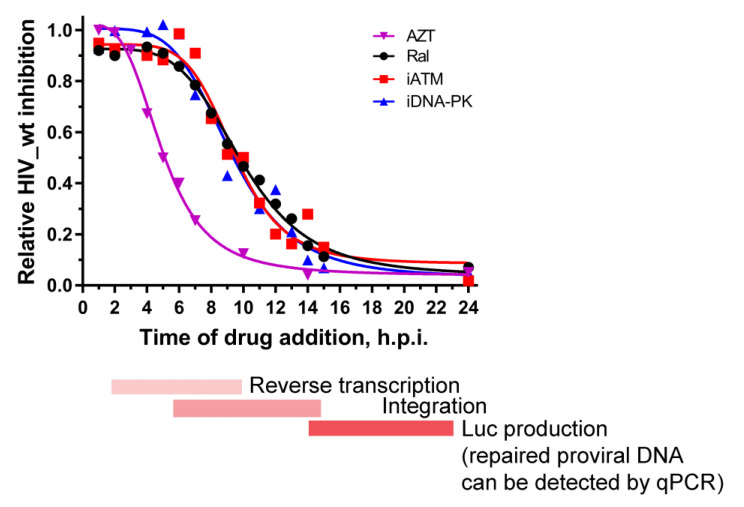
Dependence of the activity of inhibitors on the time of their addition to HIV_wt transduced cells. 293T cells were transduced by HIV_wt for 1 h, then the excess of the vector was removed; inhibitors (AZT, Ral, iATM or iDNA-PK) were added to the cells 1–24 h.p.i. Luciferase expression was tested 48 h.p.i. The mean values of three independent experiments are presented.

For this assay, iDNA-PK and iATM were added 1–24 h post infection (h.p.i.) of 293T cells by HIV_wt. AZT and Ral were used as reference compounds that mark the reverse transcription and integration processes, respectively. In our system, AZT fully inhibited luciferase expression within 1–2 h after HIV_wt addition to the cells, then gradually decreased its activity for the next 4 h and fully lost activity at 10 h.p.i (Figure 3). Therefore, the reverse transcription of the HIV_wt RNA occurred between 2 and 10 h.p.i. Ral was fully active for the first 6–7 h.p.i, but almost completely lost activity after 15 h.p.i (Figure 3). Therefore, the integration of HIV_wt cDNA took place between 7 h.p.i. and 15 h.p.i. In the case of iDNA-PK and iATM, we used relatively low inhibitor concentrations providing only about 30–40% inhibition of HIV-1, to avoid the nonspecific effects of the inhibitors. Under such conditions, iDNA-PK and iATM lost its inhibition potential almost simultaneously with Ral (Figure 3). It means that the activation of both kinases occurs simultaneously with the integration of viral DNA, and this is just the first step in PIR.

### 2.4. Activation of DNA-PKcs and ATM Is a Sequential Process

**Figure 4:**
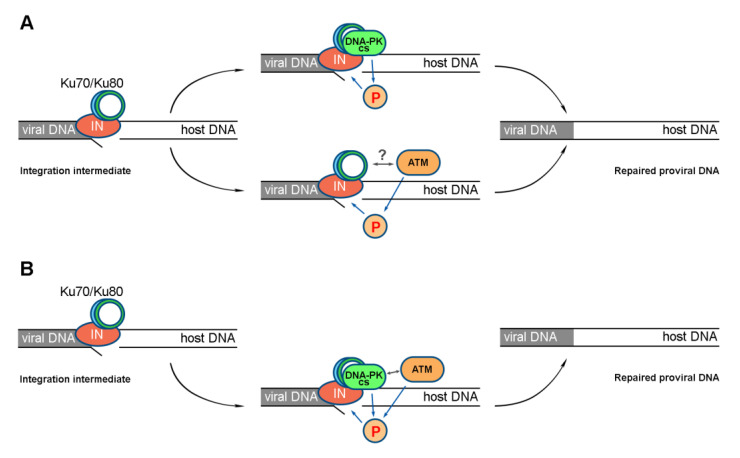
Models of ATM and DNA-PKcs activation during HIV-1 PIR. (**A**) The parallel activation model suggests that activation of either of the two kinases at the site of integration can lead to PIR activation and successful completion of this process. (**B**) The sequential activation model suggests that activation of both kinases is required for successful PIR to occur. For both models, activation of ATM and DNA-PKcs depends on the formation of the IN-Ku70 complex.

There are two possible models of ATM and DNA-PKcs activation during HIV-1 PIR. One, named parallel activation, assumes that both kinases act independently of each other (Figure 4A). The other suggests the sequential activation of kinases, when one kinase, being activated, activates another one, say, through phosphorylation, and efficient PIR is possible only in the case of both kinases’ activation (Figure 4B).

To determine which of the scenarios is indeed realized in the case of HIV-1 PIR, we studied the effect of iATM and iDNA-PK on HIV_wt, separately or in combination. If the parallel model is correct, the effect of the combination of both inhibitors will be the sum of the inhibitory activities of each drug added alone. Otherwise, the sequential model is correct.

**Figure 5:**
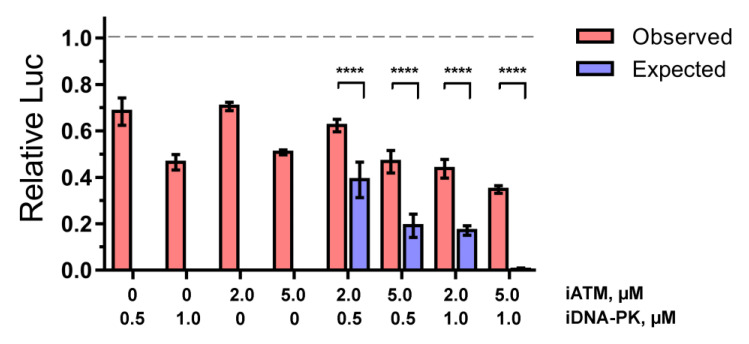
The inhibitory effect of iATM, iDNA-PK or its combinations on luciferase activity of HIV_wt in 293T cells normalized to cell viability (MTT-test). The expected inhibition efficiency was calculated for the parallel model, in which the inhibitory effect of a combination of two compounds is the sum of inhibitory effects of each individual compound. Mean values ± SD from four independent experiments are presented. Significance was determined by two-way ANOVA with Sidak’s correction for multiple comparisons, **** = adj. *p*-value < 0.0001.

HEK 293T cells were transduced by HIV_wt in the presence of iDNA-PK (0.5 or 1 μM) or iATM (2 or 5 μM) or their combinations, and luciferase activity was measured 24 h.p.i. (Figure 5). Under these conditions, the inhibitors had no significant effect on cell viability (Figure S3). Assuming that the parallel activation model is correct, we calculated the expected efficiency of HIV_wt inhibition from the combinations of both iATM and iDNA-PK as the sum of the inhibition values observed for cells treated with each of the drugs. Unfortunately for the parallel model, the expected inhibition by the combinations of the drugs was significantly lower than the values observed (Figure 5). This result rejects the parallel model in favor of the sequential model. Therefore, both ATM and DNA-PKcs regulate PIR by participating in the same signaling cascade.

### 2.5. ATM and DNA-PKcs Activation Leads to γH2AX Histone Accumulation during PIR

The histone variant H2AX is rapidly phosphorylated (denoted γH2AX) in large chromatin domains (foci) flanking double-strand DNA breaks that are produced by ionizing radiation or genotoxic agents as well as during V(D)J recombination [28]. R. Daniel et al. found that integration promotes the transient formation of γH2AX at retroviral integration sites. This result provided the first direct evidence for the association of newly integrated viral DNA with a protein species that is an established marker of the onset of the DNA damage response (DDR) [29].

We examined H2AX phosphorylation in our model. For this purpose, 293T cells were transduced by HIV_wt, HIV_mut (containing IN defective in Ku70 binding), or HIV_E152A (containing catalytically inactive IN variant IN_E152A), and the γH2AX level was measured 10, 12, 14, and 18.5 h.p.i.

**Figure 6:**
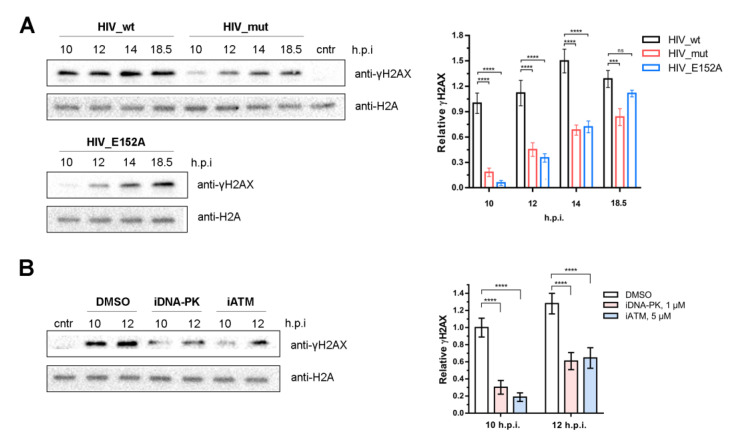
γH2AX as a marker of HIV-1 post-integrational DNA repair. (**A**) Kinetics of γH2AX formation in 293T cells transduced by HIV_wt, HIV_mut, or HIV_E152A analyzed using Western blot (left) and its quantitative analysis (right). HIV_mut contains IN with E212A/L213A substitutions defective in Ku70 binding and PIR initiation. HIV_E152A contains IN with E152A substitution defective in catalytic activity (impaired integration). The sample, designated cntr, was not transduced by any pseudovirus. Mean values ± SD of three independent experiments are presented. (**B**) γH2AX formation in 293T cells transduced by HIV_wt and treated with Nu7441 (iDNA-PK, 1 μM) or Ku-55933 (iATM, 5 μM) analyzed using Western blot (left) and its quantitative analysis (right). Samples designated as DMSO were transduced with pseudovirus but without any inhibitor, and DMSO was added to them in the same amount as to the samples treated with inhibitors. The sample denoted as cntr was treated with DMCO but not transduced by pseudovirus. Mean values ± SD of three independent experiments are presented. Significance was determined by two-way ANOVA with Sidak’s correction for multiple comparisons, ns—not significant, *** = adj. *p*-value < 0.001, **** = adj. *p*-value < 0.0001.

The formation of γH2AX was detected for all these pseudoviruses; however, the efficiency and kinetics of its accumulation significantly differed (Figure 6A). In HIV_wt treated cells, γH2AX was clearly observed as early as 10 h.p.i., reached a maximum at 14 h.p.i., and then gradually decreased up to 18.5 h.p.i. In the case of HIV_E152A and HIV_mut, γH2AX was also detected at 10 h.p.i. but at the level of 5–20% of HIV_wt, and gradually increased up to 18.5 h.p.i. (Figure 6A).

The appearance of γH2AX in cells treated with HIV_E152A or HIV_mut, where integration or PIR is impaired, respectively, can be explained by the formation of a linear non-integrated cDNA that activates DDR signaling due to the presence of free DNA ends. However, the completely different kinetics of the γH2AX accumulation in the cells treated with pseudoviruses with wild type IN and IN defective in Ku70 binding allowed us to conclude that γH2AX can be used as a marker of retroviral PIR.

Finally, we studied the role of the kinase activity of both ATM and DNA-PKcs in γH2AX formation. For this purpose, HEK 293T cells were transduced by HIV_wt in the presence of iDNA-PK (1 μM) or iATM (5 μM) and the level of γH2AX was measured 10 and 12 h.p.i. At the indicated time points, the γH2AX level was significantly reduced when ATM or DNA-PKcs were inhibited (Figure 6B). This additionally confirmed that the PIR pathway depends on the activity of both ATM and DNA-PKcs.

## 3. Discussion

Retroviral vectors are currently widely applied, both to study the individual stages of viral infection and for various genomic modifications of eukaryotic cells. In our work, VSV-G-pseudotyped HIV-1-derived vectors were used to elucidate the mechanism of repair of the cellular genome damages caused by the integration of viral cDNA, and in particular, to clarify the role of the components of DNA repair systems in this process.

To detect DNA damage sites, which are deleterious for genome integrity, and to coordinate the repair process, eukaryotes have evolved a complex signaling network, called the DNA damage response (DDR), which is principally mediated by the PI3K kinases: ataxia telangiectasia mutated (ATM), ATM and Rad3-related (ATR) and DNA-dependent protein kinase (DNA-PK), and by members of the poly(ADP-ribose) polymerase (PARP) family. ATM and DNA-PK are primarily activated by double-strand breaks (DSBs), whilst ATR is stimulated at regions of single-stranded DNA (ssDNA) generated at DSBs or stalled replication forks [30]. Viruses have developed various strategies to modulate DDR, and some of them use DDR for their own benefit. This fact gives us some new approaches to suppressing viral infection, provided that we have a good understanding of all the relationships between the virus and cellular components.

Along with other viruses, HIV-1 can exploit the DDR players to its advantage [31]. Integration of the viral cDNA results in the formation of five nucleotide gaps in cellular DNA and overhanging dinucleotides at the 5′-ends of the integrated viral DNA [14]. These unusual and serious DNA damages should be repaired; thus, the activation of host DNA repair pathways is a consequence of retroviral infection. Indeed, the participation of such DNA repair pathways in HIV-1 replication, such as base and nucleoside excision, homologous recombination (HR), and non-homologous end joining (NHEJ), has been studied using different approaches [16,17,18,19]. For example, based on the structural similarity of the integration intermediate and base excision repair (BER) intermediate, some groups suggested the participation of BER machinery in PIR [18,32,33]. For example, polymerase beta (Polβ) knock-down resulted in a decrease in HIV-1 replication, which can be considered as indirect evidence of the possible involvement of Polβ activity in the PIR process [18,33]. However, further research demonstrated that overexpression of both catalytically active and catalytically inactive Polβ restored HIV-1 replication in Polβ^−/−^ cells [34]. Therefore, Polβ is unlikely to participate in PIR. Recently, the participation of the cellular complex FANCI-D2 from the Fanconi anemia DNA repair pathway in the PIR process was assumed [35]. Based on the cellular function of the FANCI-D2 complex and the fact that the depletion of the complex proteins and downstream enzymes blocks viral DNA integration, the authors hypothesized these proteins may be responsible for the PIR process. However, a decrease in the amount of integrated DNA immediately after the onset of integration (12 h.p.i) and an increase in the amount of non-integrated 2-LTR circles in the knockout cells indicate that these proteins directly affect the integration process, rather than DNA repair after integration. To confirm their assumption, the authors need to measure the efficiency of post-integration DNA repair, using, for example, the qPCR assay described in [20].

As noted above, the first evidence for the involvement of PI3K kinases in HIV-1 replication was obtained more than 20 years ago [23] and subsequently confirmed in numerous studies [24,25,36,37]. However, the lack of specific methods for measuring the effectiveness of PIR did not allow us to study the role of each kinase and unambiguously show their participation in PIR.

The development of the first quantitative assay to measure PIR efficiency [20] allowed us to demonstrate the involvement of the DNA-PK complex in HIV-1 PIR [21]. This was a rather surprising result, given that DNA-PK is a sensor for double-strand breaks [22] that are not formed during integration. We proposed an unusual mechanism that explains this result: the key regulator of PIR is the complex of HIV-1 IN and the cellular Ku70 protein, which is part of the DNA-PK complex. The IN-Ku70 complex recruits the Ku80 and DNA-PKcs subunits, and the active DNA-PK complex is formed at integration sites. This mechanism is supported by the fact that PIR can be suppressed by disrupting the interaction between IN and Ku70 [21].

Here, we applied the same strategy that we used to elucidate the involvement of the DNA-PK complex in HIV-1 PIR to study the participation of two other PI3K kinases, ATM and ATR, in this process. We have shown that the inhibition of DNA-PKcs and ATM, but not of ATR, significantly reduces PIR efficiency, and therefore, HIV-1 exploits these two kinases, which are usually activated in response to double-strand DNA breaks, to repair unpaired overhanging dinucleotides and single-stranded gaps flanking integrated viral cDNA.

Previously, we measured the kinetics of post-integrational repair using a qPCR approach, and showed that while an integrated proviral DNA can be detected as early as 8–9 h.p.i., the amount of repaired DNA only started to increase after 17 h.p.i. [20]. This means that at least in our system, the PIR process lasts for about 8–9 h post integration. Here, we performed time-of-addition experiments to determine the kinetics of DNA-PKcs and ATM activation. Inhibitors of the different replication stages were added to cells transduced by the HIV_wt pseudovirus at different time points after the transduction, and their inhibitory efficiency was measured. It turned out that the inhibitors of both kinases lost their activity almost simultaneously with the integration inhibitor. Therefore, kinase activation occurs immediately after the act of integration. This allows us to assume that DNA-PKcs and ATM activation is a trigger for the PIR process. This correlates well with the fact that the PI3K kinases trigger repair processes of the cellular genome.

In our opinion, the most surprising result of our studies is that ATM activation requires the formation of the IN-Ku70 complex. This is not so surprising for DNA-PKcs, since during NHEJ, it is recruited to DSB and then activated due to the interaction with the Ku70/Ku80 heterodimer [22]. However, interactions of ATM with this heterodimer have not yet been reliably shown. We cannot establish that ATM interacts with Ku70 or its complex with IN. Based on our experiments, we can only state that the activation of DNA-PKs and ATM is a sequential process, when one kinase, being activated, activates the other, and effective PIR is possible only when both kinases are activated.

The need for the activation of both DNA-PKs and ATM for effective PIR was also confirmed by the rapid accumulation of the phosphorylated histone γH2AX in cells transduced by the HIV_wt pseudovirus and the significant decrease of the γH2AX level in the presence of both kinases’ inhibitors. Moreover, the kinetics of γH2AX accumulation in cells treated with HIV_wt or pseudovirures HIV_E152A or HIV_mut defective in integration or PIR, respectively, was found to be quite different. These data strongly suggest that γH2AX can be used as a marker of retroviral PIR.

## 4. Materials and Methods

### 4.1. Cell Culture and Work with Lentiviral Vectors

293T cells were obtained through the NIH AIDS Research and Reference Reagent Program. 293T cells were cultured in DMEM medium supplemented with 10% FBS and 100 I.U./mL penicillin/100 μg/mL streptomycin solution (all from Gibco, Billings, MT, USA) in a 37 °C incubator with a humidified atmosphere of 5% CO_2_ in air.

### 4.2. HIV-Based Vectors Preparation

To generate the VSV-G-pseudotyped single-round luciferase-expressing HIV-based pseudoviruses HIV_wt, HIV_mut, or HIV_E152A, 293T cells were co-transfected with HIV-1 packaging vector pCMVΔR8.2 (Addgene plasmid #12263), pCMVΔR8.2_E212A/L213A [21], or pCMVΔR8.2_E152A [21], a vector for the expression of protein G from the vesicular stomatitis virus (VSV) pCMV-VSVG (Addgene plasmid #8454), and reporter plasmid pUCHR-inLuc-mR [38] using calcium-phosphate transfection. Forty-eight and seventy-two hours post-transfection supernatants were harvested, pseudoviruses were concentrated using centrifugation at 80,000× *g* for 1 h 30 min and resuspended in PBS. The level of p24 was assayed using the HIV-1 p24-antigen ELISA Kit (Vector Best, Novosibirsk, Russia).

### 4.3. Cells Transduction

293T cells were transduced by the addition of pseudoviruses to the cell media at a final concentration of 100 pg of p24 per 10^5^ cells, which was equal to MOI = 1, calculated as the amount of integrated DNA per cell for the HIV_wt vector using gag-alu specific PCR [39]. After 1 h, the cells were treated with Nu7441, Ku-55933, AZ20, combinations of Nu7441 and Ku-55933, or DMSO as a control at concentrations indicated in the figure captions. Cells were harvested at 24 h post infection, the cell number was counted, and luciferase activity in the cell lysates was measured using a Victor X5 2030 (Perkin Elmer, Waltham, MA, USA) plate reader and luciferase assay system kit (Promega, Madison, WI, USA). The resulting data were normalized to cell count.

### 4.4. qPCR

The total and integrated vDNA were quantified 24 h.p.i. as previously described [40]. Post-integrational gap repair efficiency was measured using modified Alu-specific PCR, as described in detail in [20].

### 4.5. Time-of-Addition

To perform the time-of-drug-addition assay, cells were transduced by HIV_wt vector (100 pg of p24 per 10^5^ cells) for 1 h at 37 °C, and unbound vectors were removed by washing with 1x PBS. Then, the medium was replaced by complete DMEM. After 1, 2, 3, 4, 5, 6, 7, 8, 9, 10, 11, 12, 13, 14, 15, and 24 h.p.i., the azidothymidine, raltegravir, Nu7441, or ATM were added to cells to a final concentration of 10 μM, 10 μM, 1 μM, and 5 μM, respectively. The cells were lysed 48 h post infection and luciferase activity were measured as described in Section 4.3.

### 4.6. Western Blot

Cells were washed with ice-cold PBS, pelleted, and lysed in 50 mM Tris–HCl at pH 8.0, 150 mM NaCl, 0.5% SDS, and 1% NP-40 supplemented with a Halt Protease inhibitor cocktail (Thermo Fisher Scientific, Waltham, MA, USA) and Phosphatase inhibitor cocktails 2 and 3 (Sigma, St. Louis, MO, USA) on ice for 30 min. Lysates were then cleared by centrifugation for 10 min at 14,000× *g*. Total protein concentration was measured using a DC protein assay (Bio-Rad, Hercules, CA, USA) and 10–50 µg of protein was mixed with loading buffer. For the analysis of H2A and γH2AX, protein samples were separated on 4–15% Mini-PROTEAN^®^ TGX™ Precast Protein Gels (Bio-Rad) and transferred to a PVDF membrane using Trans-Blot Turbo RTA Mini 0.2 µm PVDF Transfer Kit (Bio-Rad). The primary antibodies used were: Histone H2A Antibody II (#2578, Cell Signaling), Phospho-Histone H2A.X (Ser139) (20E3), and Rabbit mAb (#9718, Cell Signaling). HRP-conjugated anti-rabbit antibody (Sigma) was used as secondary antibody. Immuno-reactive bands were detected on the ChemiDoc MP system (Bio-Rad) using the Clarity Western ECL substrate (Bio-Rad).

## Data Availability

Not applicable.

## Supplementary Materials

The following supporting information can be downloaded at: https://www.mdpi.com/article/10.3390/ijms24032797/s1.
