# Supplementary material for: Both ATM and DNA-PK Are the Main Regulators of HIV-1 Post-Integrational DNA Repair
Source: Int J Mol Sci. 2023 Feb 1;24(3):2797. doi: 10.3390/ijms24032797 (PMC9917498; doi:10.3390/ijms24032797)
Supplement: Supplementary file 1 [file ijms-24-02797-s001.zip › ijms-2125031-supplementary.pdf]

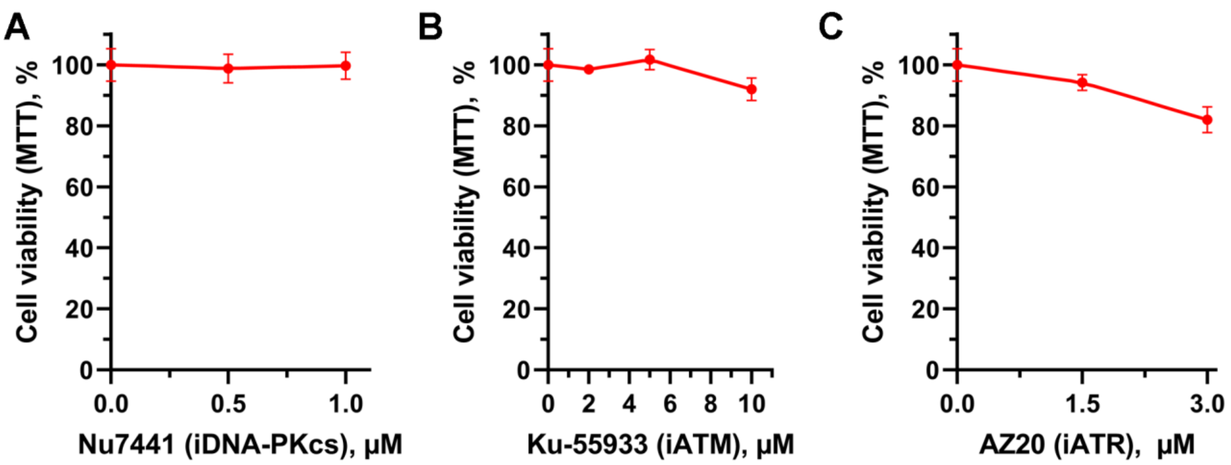

Figure S1. Cell viability (MTT-assay) in presence of (**A**) Nu7441 (iDNA-PKcs), (**B**) Ku-55933 (iATM) or (**C**) AZ20 (iATR) measured 24 hours after inhibitor addition.

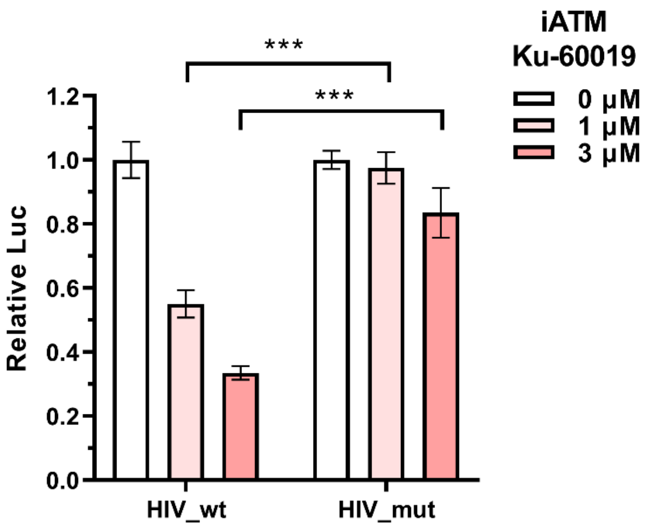

Figure S2. Effect of the ATM inhibitor (Ku-60019) on luciferase activity in 293T cells transduced by HIV_wt and HIV_mut. Mean values ± SD of two independent experiments are presented. Significance was determined by Two-Way ANOVA with Sidak's correction for multiple comparisons, ***=adj.p-value < 0.001.

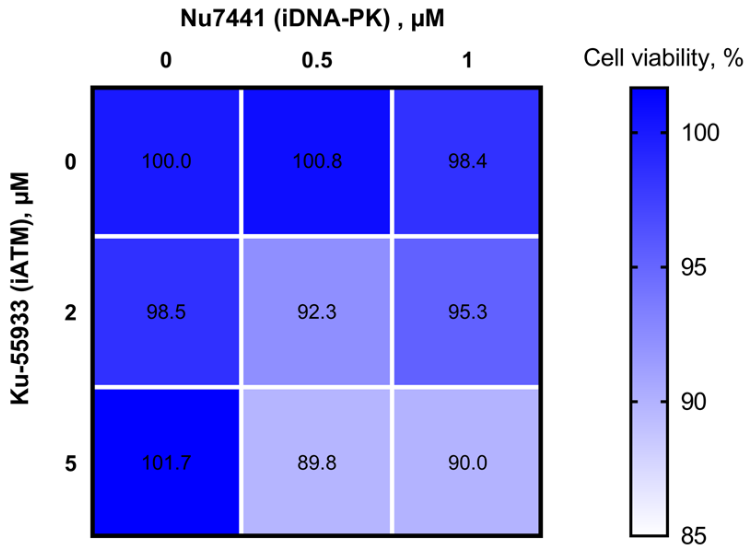

Figure S3. Cell viability (MTT-assay) in presence of Nu7441 (iDNA-PKcs), Ku-55933 (iATM) or its combinations measured 24 hours after inhibitor addition.
